# “We are also here”—Spiritual Care Practitioners’ Experiences of the COVID-19 Pandemic: A Qualitative Study from Poland

**DOI:** 10.1007/s10943-021-01492-3

**Published:** 2022-01-07

**Authors:** Jan Domaradzki

**Affiliations:** Department of Social Sciences and Humanities, Poznan University of Medical Sciences, Rokietnicka 7, St., 60-806 Poznan, Poland

**Keywords:** COVID-19 pandemic, Chaplain, Poland, Religion, Spiritual care, Spiritual care practitioners

## Abstract

Although healthcare professionals have become the symbol of risk and sacrifice during the COVID-19 pandemic, spiritual care practitioners (SCP) have also put themselves at great risk while offering their service in hospitals, hospices and other healthcare facilities. This study was designed to explore the lived experiences of SCP during the current health crisis in Poland. Semi-structured interviews were conducted with twenty-four SCP. Nine major themes emerged from the interviews: personal reactions to the pandemic, SCP’s perception of the pandemic, the impact of COVID-19 on the provision of spiritual care, spiritual needs during the pandemic, work-related emotions, the impact of the COVID-19 on religion, the role of spiritual care during the outbreak, the healthcare professionals’ perceptions of SCP and barriers to the provision of spiritual care during the pandemic. The SCP indicated that although the COVID-19 crisis has affected the availability of pastoral, religious and spiritual care, it has amplified the importance of such care and has positively influenced the visibility of SCP in modern healthcare practice. Nonetheless, in such desperate times, SCP are still neglected and should be further recognised and integrated into the healthcare system.

## Introduction

As of 29 April 2021, the COVID-19 pandemic resulted in 150,348,745 infections and 3,167,005 deaths worldwide. Similarly, despite the control measures imposed by the Polish government, after three waves of the pandemic, the morbidity and mortality rates have been rising, with 2,785,353 confirmed cases and 67,073 deaths (Serwis Rzeczypospolitej Polskiej, 2021). Such high rates, combined with physical and social restrictions, have resulted in health, economic and psychological uncertainty. Moreover, as the level of anxiety, depression and loneliness in the general population has increased, COVID-19 has caused the next global ‘pandemic’—the one of loneliness and fear (Bartoszek et al., 2020; Lahav, 2020; Malesza & Kaczmarek, 2021; Salari et al., 2020).

Indeed, ever since COVID-19 struck, apart from the biological virus, we have also been facing ‘the virus’ of loneliness, uncertainty and depression, which is particularly observable in the clinical setting, where patients are suffering and dying without the support of their loved ones or the possibility  of saying goodbye (Ferrell et al., 2020; Murphy, 2020; Olsman, 2020; Wrona et al., 2020). Although the situation in Poland is not as dramatic as in other countries, including the USA, India, Brazil, Mexico, the UK, Italy or France, which have the highest numbers of infections and deaths, still, for many patients, isolation brings more fear than the virus itself, especially as all hospitals remain closed to visitors to protect vulnerable people and avoid overcrowding of intensive care units (Dutra & Rocha, 2021).

Furthermore, 95,057 healthcare professionals in Poland were infected (22,493 physicians, 57,060 nurses, 5488 midwives, 3317 paramedics, 2536 pharmacists, 2254 dentists, 1879 laboratory diagnosticians and 30 feldshers) and 222 have died (106 physicians, 78 nurses, 14 dentists, 7 paramedics, 6 midwives, 5 pharmacists, 4 feldshers and 2 laboratory diagnosticians) (Mroczek, 2021). Consequently, the healthcare professionals have become a symbol of risk and sacrifice (Chirico & Nucera, 2020a; Pandey & Sharma, 2020; Simons & Vaughan, 2020).

However there are also spiritual care practitioners (SCP), who are trained to facilitate positive spiritual and religious coping for people who experience illness (and their families), including chaplains, priests, nuns, pastors, rabbis and imams, who have put themselves at great risk while offering their service in hospitals, hospices, long-term and elderly care facilities and other healthcare institutions. Indeed, thousands of SCP all around the world have been working tirelessly, often without proper personal protective equipment offering spiritual assistance and presence, prayers and sacraments to the diseased, their families, relatives and healthcare professionals, and many have paid the highest price for their service. Nevertheless, this is often unnoticed.

During the first wave of the COVID-19 pandemic, the world was shocked by the news of the tragic deaths of numerous Catholic priests across Italy (Bramstedt, 2020; Chirico & Nucera, 2020b). Recently, The Council of the Bishops’ Conferences of Europe announced that as a result of the pandemic all Catholic Churches in Europe suffered serious losses, as 400 priests, nuns and elderly religious died, 181 in the Netherlands, 121 in Italy, 70 in Spain, 10 in Poland, 5 in Belgium and Ukraine, 3 in Ireland and 1 in Lithuania (Polsat News, 2020).

This is important because although the COVID-19 pandemic has brought spiritual care to the forefront of healthcare and there is growing evidence for the positive role of SCP in the healthcare system during the current healthcare crisis (Busfield, 2020; Cadell et al., 2021; Drummond & Carey, 2020; Dutra & Rocha, 2021; Ferrell et al., 2020; Harrison & Scarle, 2020; Jones et al., 2020; Koenig, 2020; Murphy, 2020; Papadopoulos et al., 2021; Peacock, 2020; Sarmiento, 2020; Swift, 2020; Weinberger-Litman et al., 2020; Wierstra et al., 2020), little is known about the impact of COVID-19 on spiritual care in Poland.

This study explored the experiences of SCP on the impact of the coronavirus outbreak on the provision of spiritual care in Poland. While assessing SCP’s experiences of the pandemic, this paper considers how COVID-19 functionally affected the provision of spiritual care and whether it has increased its importance. It also describes how SCP have responded to the pandemic and how they have perceived their role during the crisis. Finally, it examines whether the status of the SCP as key healthcare workers has been recognised.

## Method

### Study Design

As there  was no other research on the experiences of SCP during the COVID-19 pandemic in Poland, this qualitative study (Creswell & Porth, 2017) involved semi-structured interviews with twenty-four SCP who provided spiritual care during the pandemic in Poznan, Poland (Appendix). The study focused on SCP’s lived experiences, so an interpretative phenomenological approach was adopted (Crowther et al., 2017; Peat et al., 2019) to understand the meaning participants gave to their experiences and provided new information about how these meanings influenced their choices, thereby providing in-depth knowledge about the spiritual care provided during the COVID-19 pandemic in Poland from the perspective of SCP.

The study was performed in line with the principles of the Declaration of Helsinki, and according to local legislation and national guidelines on research involving human subjects, ethical approval was not required. However, because some participants asked for such approval, it was obtained from the Poznan University of Medical Sciences Bioethics Committee (KB—51/21). Additionally, all participants were informed about their right to quit the interview at any given moment or not to reveal information regarding their personal circumstances.

### Recruitment and Data Collection

Semi-structured interviews were conducted with twenty-four SCP who provided their service within a healthcare setting during the COVID-19 pandemic. Eligible participants were recruited via hospital websites and received either a telephone or email invitation to participate in the study. They were included if they were SCP, directly involved in the spiritual care within a healthcare setting during the pandemic and willing to participate in the study. Thirty SCP responded, but six did not participate because of a lack of time (*n* = 4), previous negative experiences giving an interview (*n* = 1) and unwillingness to discuss personal experiences (*n* = 1).

SCP gave informed consent and were scheduled to be interviewed between November 2020 and March 2021. However, due to safety reasons, twenty-three interviews were conducted via the telephone, lasting between 30 and 65 min, and one was performed as a written interview. All interviews were audio-recorded and transcribed verbatim by the author. For analytical purposes, any emotions, intonations, silences or emphases were also transcribed. Only two participants asked for the authorisation of the transcribed interview.

### Data Analysis

The interview transcripts were coded, read and analysed using thematic analysis (Guest et al., 2012). To conceptualise participants experiences, Colaizzi’s (1978) seven-stage phenomenological method was used for the analysis. First, each transcription was read several times to obtain a sense of the participant’s experiences and identify the thematic themes. Second, once all the transcripts were read, the identification of significant statements, phrases and key words that directly related to the phenomenon under investigation has been performed. Third, after reviewing and comparing these significant statements, they were consolidated to reflect the participants’ meanings. Fourth, these statements were organised into thematic clusters that were integrated into one of nine major themes identified. Fifth, an exhaustive description of all theme clusters and associated formulated meanings were developed. Sixth, all the themes were defined and presented in the form of an explicit and clear statement. The final stage aimed to ensure that SCP’s intended meaning was conveyed in the fundamental structure of the phenomenon. Thus, in order to validate the essence of the phenomenon, a follow-up appointment was made with or the transcript was sent to the participants for any additions or deletions and, if necessary, alterations were made according to their feedback.

## Results

### Participants

A total of twenty-four SCP were interviewed (mean age: 49.08; range: 36–67; mean years of experience: 13.8; range: 3–40), of which, twenty-two were Polish, one was Polish-American and one Polish-speaking Moroccan (see: Table 1). All respondents were males and lived in large agglomerations. They had the following religious affiliations: Roman Catholics (*n* = 19), Lutheran (*n* = 1), Muslim (*n* = 1), Judaism (*n* = 1) and Jehovah’s witnesses (*n* = 2). Seventeen worked as hospital chaplains (*n* = 8 full-time; *n* = 9 part-time workers) and other settings (*n* = 7), including a hospice (*n* = 1) and community settings (*n* = 6).

**Table 1:** Participant demographics

Respondent’s code	Denomination	Position	Gender	Nationality	Age	Years of experience	Place of service
CC1	Roman Catholic	Priest	Male	Polish	36	5	Provincial hospital
CC2	Roman Catholic	Priest	Male	Polish	67	32	Clinical hospital
CC3	Roman Catholic	Priest	Male	Polish	67	27	Clinical hospital
CC4	Roman Catholic	Priest	Male	Polish	66	8	Clinical hospital
CC5	Roman Catholic	Priest	Male	Polish	38	7	Clinical hospital
CC6	Roman Catholic	Priest	Male	Polish	60	8	Clinical hospital
CP7	Roman Catholic	Priest	Male	Polish	37	6	Clinical hospital
CC8	Roman Catholic	Priest	Male	Polish	41	12	Provincial hospital
CC9	Roman Catholic	Priest	Male	Polish	45	10	Provincial hospital
CC10	Roman Catholic	Priest	Male	Polish	58	23	Clinical hospital
CC11	Roman Catholic	Priest	Male	Polish	61	27	Clinical hospital
CC12	Roman Catholic	Priest	Male	Polish	37	7	dedicated COVID-19 hospital
CC13	Roman Catholic	Priest	Male	Polish	41	12	Clinical hospital
CC14	Roman Catholic	Priest	Male	Polish	38	5	dedicated COVID-19 hospital
CC15	Roman Catholic	Priest	Male	Polish	39	7	Community service
CC16	Roman Catholic	Priest	Male	Polish	61	14	hospice
CC17	Roman Catholic	Priest	Male	Polish	37	6	dedicated COVID-19 hospital
CC18	Roman Catholic	Priest	Male	Polish	41	8	Clinical hospital
CC19	Roman Catholic	Priest	Male	Polish	54	15	Provincial hospital
EA	Evangelical	Pastor	Male	Polish	39	8	Community service
R	Jewish	Rabbi	Male	Polish-American	65	40	Community service
I	Muslim	Imam	Male	Moroccan	59	20	Community service
JW	Jehovah’s Witness	secular	Male	Polish	55	22	Community service/hospital
JW	Jehovah’s Witness	secular	Male	Polish	36	3	Community service/hospital

All but one of the participants lived in the city with over 500,000 inhabitants

### Themes

**Fig. 1:**
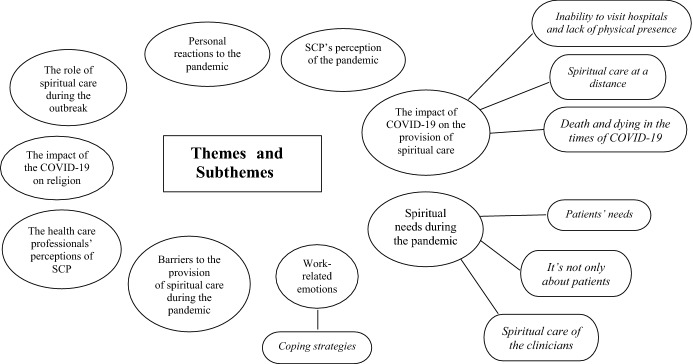
Themes and subthemes emerging from qualitative analysis

The analyses of the interviews identified nine major themes with associated sub-themes: 1. personal reactions to the pandemic, 2. SCP’s perception of the pandemic, 3. the impact of COVID-19 on the provision of spiritual care, 4. spiritual needs during the pandemic, 5. work-related emotions, 6. the impact of the COVID-19 on religion, 7. the role of spiritual care during the outbreak, 8. the healthcare professionals’ perceptions of SCP, 9. barriers to the provision of spiritual care during the pandemic (see: Fig. 1).

## Theme 1: Personal Reactions to the Pandemic

When asked about their first reactions after hearing about the COVID-19 outbreak, SCP evoked a wide range of emotions and reactions, but all described their feelings of insecurity, frustration or anger. Although they emphasised the negative impact COVID-19 has had on society, the healthcare system and their everyday routine, SCP were more concerned over their inability to provide pastoral and spiritual care than being infected. Such feelings resulted from their concerns that due to the social restrictions they would not be able to fulfil people’s spiritual needs. Moreover, while being disorientated and confused, some felt frustration and complained that even in the hardest of times churches have previously never been closed.

At the beginning, it was anger and irritation caused by the closing of the churches and society. It really hurt me when I saw that fewer people attended services. (EA)

I didn’t realise what was going on. I didn’t understand why they were closing us down and whether all these restrictions in hospitals were necessary. I am still very concerned because even in the hardest times, during the past epidemics, churches weren’t closed and priests weren’t restricted from visiting the sick. (CC9)

Also, participants who feared the virus were not so much afraid of contracting the disease rather felt a responsibility for not putting patients or parishioners at risk.

I didn’t want to be infected as I could pose a threat either to patients or I could bring something back to the parish. I was anxious about it. (CC10).

At the beginning, I was scared of how would my parishioners react to the fact that I am working in the covid hospital (CC12)

Some respondents described their reactions from a theological perspective, for example, two Jehovah’s witnesses stressed that although they did not perceive the pandemic in terms of “God’s intervention” or “punishment”, it was not a surprise as it was somehow anticipated.

The pandemic was a surprise, nobody expected it. (…). However, the disease itself did not surprise us, because its prophecy appears in the Bible (…) we believe that such things will happen. Thus, while the pandemic has brought some unexpected consequences it was not a big surprise. (JW1)

Similarly, an older Catholic priest who had experienced a pulmonary embolism, while referring to chaplaincy as “a vocation”, also suggested that while he might have used the governments’ restrictions as an excuse for his service in the hospital, he emphasised that pastoral and spiritual care was his calling and pastoral duty.

I am a sick person, I have undergone a pulmonary embolism, so I am at greater risk. I could have used the director’s decisions and restrictions as an excuse. I could have just sat here and wait for a call. It was my personal decision to go and visit the wards asking whether anyone needed spiritual care (…). Physicians, nurses and patients often ask me if I was not afraid. I reply that although I am afraid, I am also present. I just cannot run, desert or say that I couldn’t come. (CC4).

## Theme 2: SCP’s Perception of the Pandemic

While describing their experiences of the pandemic, most SCP were moved by the alarming rates of loneliness and depression in society. In particular, they suggested that while being sick in the hospital is always difficult, it became even worse during the current health crisis as most patients were alone on the wards; thus, the participants complained that many patients feel abandoned, are suffering and dying without the support of their loved ones and the possibility to say goodbye.

When I entered the covid ward for the first time, I was shocked at how lonely the sick must feel. Being sick is always a very difficult experience. There is always uncertainty, anxiety, fear, the unknown environment, but visits are possible and they make it much easier. And now, due to covid, the feeling of loneliness is very difficult. (…) Especially the elderly are so confused. Moreover, sometimes they are not able to use their phones. So, the saddest thing for me was that many people are so alone there, and sometimes are also dying in loneliness. (CC12)

However, some SCP remained stoical and argued that although the situation was different, they did not find it more difficult or problematic than before the crisis. For example, both a rabbi with forty years of experience and a Jehovah’s Witness perceived the pandemic more as a “challenge” than a threat or problem.

Personally, I feel fine with it. For me, it is rather a different, not a difficult, situation. I would say it is a challenge, not a problem. (R)

It is more of a challenge. (JW2)

Some suggested that as hospital chaplains they experienced risk, uncertainty, suffering and death daily and were somehow used to it, while others rationalised the situation by arguing that people also had to survive in the past and adapt to the horrific reality of war which became their “normality”.

I would describe it as normal life in slow motion and there are much fewer people everywhere. (…) This is our new ‘normality’. People who are sick, suffering or dying experience it day by day, night by night; this is their life. (CC1)

Of course, it cannot be compared, but people who experienced the horrors of concentration camps in Dachau or Auschwitz also had to survive. They had to live somehow. It was their horrific ‘normality’. (CC14).

Interestingly, while SCP stressed how the COVID-19 has negatively affected their routine and the possibility of providing spiritual care, some acknowledged its unintended, positive consequences. They suggested that the pandemic has made people appreciate everyday life more, helped them to realise the true meaning of life, taught them new skills and made them familiar with new technologies.

Although after a year people are tired, I also notice that they appreciate everyday life more and all the little things, like sunshine. (CC15)

What is somehow positive is that due to the quarantine and isolation more people have experienced what it means to live like our parents or grandparents who often cannot leave their homes; what it means to live in social isolation. (CC17)

It is hard to admit that there is something positive in the pandemic, however (…) it has helped us all to accustom ourselves to new technologies, especially the seniors (…) they have overcome their resistance and are now doing great. And it motives us to use these tools. (JW1)

Finally, SCP reflected on how the outbreak has affected their perception of spiritual care and emphasised that the pandemic gave them extra motivation to serve those in need.

Because many wards are closed to visits I cannot enter everywhere. However, I have more time for other activities: I can bring patients something from their families or talk to them a bit longer. (CC4).

I am very happy that I can do my service. The more I visit patients, the more motivated I am. I felt I just couldn’t come. I cannot be scared (CC8).

The pandemic made me realise even more that the sick, not only Jehovah’s witnesses, need consolation (…) This is my most important reflection, and it motivates me to do my service the best I can. (…) Thus, I do not experience any type of discouragement. On the contrary, I feel motivated because I see positive effects stemming from the Bible and the hope it gives people. (JW1)

## Theme 3: The Impact of COVID-19 on the Provision of Spiritual Care

All the SCP interviewed emphasised how COVID-19 has negatively influenced their ability to provide spiritual care. They complained that because of the outbreak, spiritual care was often served only on-call or when they were present in the ward and that they did not have enough time for conversations with the patients and were unable to celebrate the mass. Others suggested that due to sanitary restrictions they had problems with administering the sacraments or had to limit their service.

As hospital chaplains we often work, you know, ‘hurry, hurry’ in a big rush, as we have to visit many patients on many different wards. So we often simply administer sacraments, but patients would like us to sit with them for a moment and have a talk. But because of covid, it is impossible to go there and talk with everybody for, say, half an hour. We do not have time for that. (CC3)

My time in the dirty zone is restricted from two to four hours at a time. It results from the great fatigue and exhaustion caused by working in the uniform. Consequently, conversations with the patients are much shorter. Thus, if there are no special needs, I simply administer the sacraments, especially the penitence, the Eucharist and the anointing of the sick, so I can visit more patients. (CC7)

The pandemic has greatly affected my work. I serve both in the covid hospital and in my parish where I visit the sick once a month. At the beginning, I didn’t visit them at all, as the archbishop suggested we should withhold the visits. Later we came back to it, but me personally, I limit those visits to a minimum. I mean, to ordinating the communion, I just don’t have… I mean, I have decided not to take a risk, for the sick, I don’t want the risk their health. If I can bring them something, I do it, but I do not talk to them a lot. I ordinate sacraments, two or three words and that’s all. Also in the hospital, there is no room for spiritual conversation, it is rather the ordination of sacraments, especially to those who are unconscious. (CC12)

### Inability to Visit Hospitals and Lack of Physical Presence

While all the SCP reported that COVID-19 had seriously limited the possibility of providing spiritual care, it was especially non-Catholic ministers who complained over not being allowed to enter healthcare facilities and care for the patients. Simultaneously, they emphasised the importance of “being” and described physical presence as a fundamental dimension of spiritual care.

What is new is that I cannot meet people personally in hospitals or care homes. I haven’t been in the hospital for almost a year. Because I cannot visit hospitals I talk to them [patients – JD] only by phone. (R)

From the very beginning, I haven’t been allowed to enter medical centres. It is impossible to have any contact with the sick. I cannot visit them. (I).

The possibility of providing spiritual care has been restricted. I deeply regret it because physical presence is essential to spiritual care. (EA)

However, one Catholic priest claimed that the pandemic has turned the entire healthcare system upside down. Others complained that the sanitary restrictions (limitation of visits, disinfections, masks and protective uniforms) have caused huge disorganisation in the provision of spiritual care.

It is different. I cannot enter two wards and many others have set their own rules. (…) I have received instructions from different wards, even for confession, and a confirmation that after fulfilling these recommendations and restrictions I could do my service normally. (CC4).

The pandemic has changed the entire structure of the hospital. It has been divided into “the clean area”, where the medics stay and where the chapel is, and “the dirty area”, where the patients are placed. The entrance into the dirty area is possible only in the protective uniform (coveralls, the mask and the shield, gloves and the goggles). As a chaplain, I had to undergo special training sessions in working in such a uniform and making contact with patients affected by infectious diseases. (CC7)

Others complained that patients often felt confused because they did not recognise the chaplain.

The sick call me jokingly ‘an alien’. At the beginning, they were scared a bit, but they got used to it. (CC13)

[a]ll the personnel look alike, they all wear the uniforms, so one cannot differentiate between people. Especially the elderly are confused. They cannot recognise people and because of the masks and helmets you cannot hear others properly. (CC19)

Thus, SCP complained not so much about the restrictions but the way they affected their ability to provide spiritual care. Moreover, as the number of SCP in hospitals was reduced, participants found it difficult to meet all patients and fulfil their spiritual needs. Consequently, they argued that it was lack of physical presence and shortening of time for conversation with the patients that bothered them the most.

The hardest thing is the shortening of the time of conversation with the patients and healthcare professionals. I also miss the help of volunteers who helped me in my service. (CC2)

Even when I can enter the hospital, they do not let me everywhere. I am not allowed to visit oncology wards and other ‘covid wards’. It is forbidden. (…) In other locations, patients can leave the ward but on such occasions, there is neither much privacy, as we meet in groups, nor time. However, it means a lot for them and many patients come. (CC5).

### Spiritual Care at a Distance

While emphasising the burden placed on the SCP by the pandemic, they emphasised that the biggest challenge they faced was how to “show up” for the patients’ and the clinicians’ spiritual needs when physical distancing was crucial for those entering public spaces.

We cannot meet each other. This is a big change and a challenge. (…) we just had to find new means. We are now using other methods which help us to contact those who need it (…) Without the phone it would be very difficult to help the sick. (…) and videoconferences are also very useful as they allow us to see each other. (JW1)

Very quickly we have switched to the provision of spiritual care via telephone: we have set a group of people responsible for it and have informed our community about alternative means of communication, either by email or telephone. It is of key importance for the elderly and coronavirus convalescents who use it a lot. (EA)

Thus, although all denominations in Poland have either suspended or limited their religious life in the community-based dimension, all the SCP interviewed maintained contact with their believers, including those in hospitals, and adapted to the remote pastoral and spiritual care by using modern technologies, such as online-based spiritual counselling and prayers.

I can still use alternative means of communication. Sometimes I use electronic devices. I also use the telephone a lot. (CC12)

Because I have been using modern technologies to contact people for many years I have shifted towards online-based meetings. (CC15)

Participants often emphasised how electronic devices, the new media and videophone software programs have helped them to maintain contact with their communities and provide spiritual care to those who are isolated, and the sick in particular.

I have switched to online-based meetings and have transferred my lectures to Zoom. I also preach on Facebook, two times a day (…). Each day I have one-two hundred followers, and sometimes even a couple of thousands. People tell me it helps them a lot and it makes me very happy. (R)

Each Sunday I give virtual religion classes via Skype, Zoom or Google Meets. (I)

### Death and Dying in the Times of COVID-19

Although SCP, particularly hospital chaplains, admitted being accustomed to death and dying, they all declared that the COVID-19 influenced their perception of people passing away. While stressing that during the pandemic death has entered hospitals at unprecedented rates, SCP reported that death has become much more common and closer due to higher rates of severely ill patients. Thus, as eyewitnesses, they often complained how difficult it was to confront multiple concurrent deaths, moral distress and the associated feelings of helplessness. Moreover, some suggested that these distortions in the process of death and dying also affected the process of family grieving.

My hospital has always been a place where the most severely ill patients have been treated, so being with those in hard conditions or dying wasn’t new for me. However, what makes me suffer is that people are now passing away without the presence of their loved ones. I can feel their suffering even more now. I also know it is very hard for the family. It made me realise how important it is to pass away while having someone next to you. (CC7)

Death has become much closer. Its experience is much more common: many of us have witnessed it and know someone who passed due to the virus. There are so many deaths caused by it, so many serious transitions of the disease. This is a really hard experience. (CC18)

Some chaplains described how this opportunity to confront the reality of death has affected their perception of the fragility of life, the passing away and the importance of honouring the life of the deceased.

What I notice among all of us is the increasing awareness of the fragility of life (…) Yes, it affects our awareness of the passing of life, the insecurity and fluidity of plans. (CC6)

It affects my thinking about the transition and passing away. (CC15)

## Theme 4: Spiritual Needs During the Pandemic

### Patients’ Needs

Regardless of their access to hospital wards, all the SCP emphasised that the sick, and those in hospitals, in particular, feel much more isolated and that the COVID-19 has significantly increased patients’ anxiety, depression and fear. Some even suggested that it is not the virus itself that poses the biggest threat but the feeling of loneliness resulting from quarantine and isolation. Consequently, SCP stressed the role of spirituality and religious communities in supporting patients’ needs.

The coronavirus has affected people’s needs as they feel more lonely now. They lack their religious community and rituals. I often hear that they are afraid of loneliness. This is the hardest thing because they experience it a lot. Families cannot visit them and they feel much more isolated and lonely and have a deeper need for physical contact and conversation. (R)

Because physical contact is ‘forbidden’, isolation and distance are very difficult for them. Some patients have fallen into a kind of depression. They desperately need someone to visit them, to be with them or who just says ‘good morning’. During my visits, I have experienced many reactions, from tears to laughter. They seek consolation, comfort or simple human warmth, especially that, as they tell me, even their families are scared of ‘bringing something’. It’s like a death sentence. For many, it is very horrifying (CC4).

The hardest time was that of Ramadan, which was in April. Because during Ramadan we meet daily for evening praying, the meal and talk, most fellow Muslims told me it was the hardest time for them. Our community helps them in coping with fasting. They missed our community, meetings in our mosque and mutual praying. Some have even called me and insisted on my opening it, but I tried to explain to them that we also get closer to God by not putting others at risk and suggested that we should wait until the epidemic ends (I)

Thus, SCP complained that due to sanitary restrictions patients’ spiritual needs cannot be fully met. Consequently, they used their experience, communication skills and new technologies to support patients and their families isolated in care homes or hospitals, trying to fulfil their spiritual needs and assisting them with praying, sacraments or simply by listening to their problems and anxieties or helping with shopping.

Patients mainly expect a conversation about their everyday problems, their diseases or passing away. Sometimes they ask me for support from the Caritas (…) I do some small shopping or bring them things from their families. Sometimes I also help patients with contacting their families. (CC7)

Some patients miss visiting the church and sacraments. Because I cannot enter some wards, I can see patients only through the glass and give them a blessing at the front of the Holy Sacrament or absolution (…) Because I cannot enter behind the red line, nurses tell patients that they can receive general absolution (…). And many want to receive it. This is very positive. I see a great grace in it. Even though I cannot be with them and administer the anointing of the sick. (CC1)

### It’s Not Only About Patients

While caring for patients, SCP reported that the families also live in great uncertainty, feel anxious about the health risk, quarantine and suffer in silence from not being able to be with their loved ones isolated in hospital. While stressing their need for spiritual support, counselling or guidance, participants reflected how emotional families get when asking them to visit their relatives. Moreover, while reflecting on how families seek peace and help, the SCP emphasised their role as “intermediaries”, which filled them with joy and great satisfaction.

I remember an older lady whom I visited a couple of times. I also administered her the sacrament of anointing of the sick and whispered into her ear that her daughter sends her greetings, and later the lady died. That daughter was so grateful that once she came to my parish just to thank me in person. She said it meant a lot to her because they hadn’t any contact with her mother and I was the only person who could see her. (CC12)

Families and relatives ask me to pass something, even a simple bottle of water, or something I can buy in the cafeteria. Of course, patients are well-cared for, but such small gestures mean a lot to the families. Even when I bring a patient water and tell him or her that it is from their daughter or husband. They also ask for praying or the holy mass for their sick relatives. (CC19)

### Spiritual Care of the Clinicians

While focussing on the sick and their families, SCP emphasised that as a group, healthcare professionals were at increased risk and also required spiritual support in dealing with the fear, loneliness, uncertainty and constant pressure.

For me, spiritual care covers also the personnel. They also need visits and conversations. Thus, I support them, bring them hope during isolation from their families and assist in their problems and fears. (CC9)

As for the medics I support and talk to them about their problems. I help them in coping with the anxieties caused by the pandemic and bring them hope for a better future. I also administer sacraments and spread the biblical message about working with the sick, you know, the parable of the good Samaritan. (CC18)

Unsurprisingly, all participants believed that supporting the need of the healthcare professionals was the key aspect of their role as SCP. They reported that often physicians or nurses approached the chaplain to ask for help in managing their emotions resulting from work overload, emotional separation and longing for their loved ones or mourning after someone died.

Two or three physicians confessed that they suffer a lot from separation from their families. As physicians, they belong to the group at increased risk and they had to rent separate apartments. They were afraid of the possibility of infecting their loved ones. They hadn’t seen their families for a long time and missed their wives and children. (…) I tried to counsel them. I also quoted them some Quranic *verses and offered prayers.* (I)

Also, members of the medical team come to me. I serve them with conversation, talk, support and the sacraments. And when a member of the staff dies, a physician or a nurse comes to me and asks to celebrate the mass. Sometimes entire wards attend such a mass. (CC6)

Participants emphasised that healthcare professionals need to share their experiences and talk about difficult emotions and that even small gestures or a simple conversation help them to forget about the difficulties of their everyday struggle with the virus.

My contact with the clinicians is always very moving. Physicians and nurses also need compassion and conversation. Even asking a simple question ‘How do you feel?’ or ‘It’s good you are here’ means something, especially for overloaded nurses. They really appreciate my presence. I remember when once because of the quarantine there was only one nurse left on the ward, and she was very moved when I asked her how she was and how she managed to handle the situation. She was very moved that anyone cared for and worried about her. She felt appreciated. Thus, our presence gives them certainty, that they also have to be there. And it is very difficult: the risk, many tasks, including a lot of bureaucracy, visiting and caring for patients. They also suffer a lot. She told me she needed such care. (CC4).

One surgeon came to me and asked for a talk. He said he had problems with expressing his emotions. I remember his words. He said: “I feel overwhelmed by what is going on, by the number of the sick and by the fact that we can save only a few patients’. He also felt burdened by the fact that due to restrictions many patients had problems with being admitted to the hospital. (CC7)

## Theme 5: Work-Related Emotions

Although all SCP stressed how important it is to provide spiritual care during the COVID-19 pandemic, they confessed that experiencing the challenges caused by the outbreak was a difficult experience. While talking about their experiences, they emphasised how their service in hospitals has put them at risk of increased distress which resulted in intense feelings of sadness, anxiety, fear or frustration. However, they often stressed that these negative emotions resulted from the awareness that many patients were suffering and dying alone.

The saddest thing is that so many people experience loneliness and that they are also dying alone. (CC12)

During the last half of year, many people I have known have died… [moment of silence] although two persons died for other reasons than covid, it was still a difficult experience. And then… [silence] I witnessed the death of several priests. (…). In October I had a week when each day someone died. Once, four persons I knew in person were in the ICU and they all passed away. It was the hardest experience [silence]. Yes. (CC18)

While the intensity of stress was mainly related to SCP’s experience of death, most participants also suffered from loneliness and reported how isolation and lack of social contact negatively affected their emotions.

The most difficult for me is the lack of physical contact with my fellow Muslims. Especially during the month of fasting, Ramadan, I had deeply experienced the lack of our community. I really missed our evening meetings. Even though I practised at home with my children, it was not the same. I miss my community, my fellow Muslims and our communal prayers. Sometimes I even felt like being in prison. (I)

Because I work both in my parish and in the dedicated COVID hospital, I decided to isolate myself from all types of social contacts (…). For almost a year I had very few social contacts, even such as having a coffee or tea with someone. I am usually all by myself, walking alone, wearing a mask. For the very first time, I have even spent Christmas Eve alone, without my parents. So this is hard for me [loud sigh]. (CC14)

The hardest thing is the feeling of loneliness and that there are much fewer social contacts. While you can motivate yourself and handle it for a month or two, when it lasts for a year and the contact is very rare, there is no touch, no proximity no hugging, it affects people a lot, including myself. It affects my psyche and functioning (CC16)

While some participants complained of great fatigue and exhaustion caused by long working hours in their uniforms, others experienced moral stress which resulted from a major dilemma: risking the transmission of the virus to others as asymptomatic carriers, and hesitation whether the SCP needs to be physically present. Consequently, some participants experienced moral remorse resulting from such dual responsibilities and emphasised how torn they felt between their obligation to serve the needs of the sick and the responsibility of not putting them at risk.

The hardest thing for me is the dilemma I face when people call me and ask for a meeting or communal prayer. Because exposing someone at risk is a sin in Islam, I feel torn between my will to help them and my responsibility of not putting them at risk. (…) It was very stressful to me, as each time I refuse visits I constantly wonder whether I have done the right thing. I am aware that as an imam I have responsibilities towards my fellow Muslims. At the same time, my responsibility is also to protect them. I often wonder if a telephone conversation is enough. It’s very hard for me. (I)

During the first wave, there was a lot of anxiety (…). I was also aware that I could bring the infection to the hospital, so I always keep up all the safety measures and follow the instructions. Moreover, because I am also a parish priest I fear that I could infect somebody. (CC13)

Other participants felt guilty about not being able to visit all patients. Even though they were aware that it resulted from their fatigue and work overload, still they blamed themselves for not trying hard enough.

The most difficult part of my service is that I cannot reach the patients as often as I wish and I am not able to devote more time to them. As a chaplain, I am alone on my duty. I have approximately 420 patients. Although there is also another chaplain, we cannot meet each other as we are working shifts. No trained priest is allowed to enter into the infectious zone and visit patients. Thus, it is also fatiguing. (CC7)

I have this feeling that I could give more from myself… [silence]. However, due to many obligations I simply lack the time, but also strength. I am simply so tired…[silence]. I feel ambivalent about it, because while I wish more people called me for service, so I could be with them, I am also very tired and feel tension, if you know what I mean. (CC5)

### Coping Strategies

Unsurprisingly, the intensity and insecurity of the SCP’s work during the pandemic, the necessity to adapt to the sanitary restrictions, limitation of visits, the duty to work in the protective equipment, ubiquitous suffering and death, accompanied by the isolation and all the negative feelings caused by the pandemic, resulted in stress overload. As one chaplain said:

During the first months, I didn’t realise how stressed I was. It is only just now that this occurs to me and I am looking for ways to cope with all the stress I have experienced (CC11).

While describing the coping strategies they have developed during the pandemic, most SCP pointed to the praying and community support, including that from the archbishop. However, there was a kind of imbalance between the stress and their coping strategies as all participants had to cope by themselves without the mentioned professional support.

From the very beginning, I was supported by our archbishop who called me a couple of times and asked how I was doing. It was a big support. (CC6)

I cope with the stress by sharing my experiences with my friends from the community. Praying and adoration are of great help too. (CC7)

I read and talk a lot and, if possible, I try to meet people. However, it is mainly personal contact with the Lord. This is my strength. However, I am aware that God often reveals himself in other persons, so when there are no other people it is hard… yeah, that’s how I cope. (CC16)

Some priests also described their service from a theological perspective as a “sacrifice” and emphasised that it is their faith in divine intervention that helps them to maintain their duty.

It is not simple but I take it how it is. This is the sacrifice that I make during my service. If it all were so simple and super, where would be my sacrifice? (CC1)

I have this sensation that God is above all of it. He keeps watch and I can only do the best I can. Personally, I am not afraid. I am not scared of being infected. I am just doing my job. I only hope that I haven’t infected anybody. (CC9)+

Others pointed out religious significant others, either famous religious persons or Jesus himself, who served as role models that they tried to follow in times of hesitation.

I cannot be scared. I have read about the Polish Jesuit Antoniewicz who served the sick during the cholera epidemics in the 19th century and who died of cholera. I also knew Dr Wanda Błeńska, who worked in leprosy villages in Uganda and she had never refused to touch the sick. They are all great examples to all of us. Also, Jesus never refused to touch the sick. (CC4)

## Theme 6: The Impact of the COVID-19 on Religion

Although all SCP were concerned over the health crisis caused by the outbreak and stressed how it negatively affected the provision of spiritual care, they were also preoccupied with the impact of COVID-19 on religion. Simultaneously, they perceived the pandemic as a double-edged sword that might become both a religious opportunity and a threat.

In relation to spirituality, it [the pandemic – JD] may serve either as a chance of getting closer to God or it may deepen spiritual problems. (CC12)

While stressing that due to the restrictions fewer people visit churches, attend services, use pastoral guidance and sacraments, SCP defined the pandemic as a threat and emphasised how COVID-19 has negatively affected people’s religiosity. Some even worried that religious life may somehow vanish or “be cancelled”. They also considered that the pandemic has created a paradox, while religion and spirituality are important resources that help people cope in critical situations, these have been limited by COVID-19 and believers have been left without spiritual assistance.

People have been left on their own. Although alternative forms of transmissions emerged, people’s contact with spirituality has been seriously restricted, either by the temporal closing of the churches, limitation of the number of believers during the service or restriction to visits. (EA)

There are very few people in the hospital chapel (…) Before the pandemic many people who came from out-of-town attended the mass. Now they are gone. (CC1)

Consequently, SCP were concerned that COVID-19 may result in “spiritual havoc”.

Since March our [Muslim – JD] centre has been closed and we cannot pray together, even during the Friday’s prayers, which for Muslims is the most important. (I)

Because of the “imprisonment” in homes or the hospital, people cannot go to the church or meet their priest. They are left helpless and separated from the sacraments. Some confess that they haven’t had access to confession or communion for over a year and for many it is a source of suffering. I am concerned that it may result in spiritual havoc (CC4).

Thus, SCP emphasised that the pandemic has launched negative processes within the church, religious indifference and privatisation of religion:

Because of all the restrictions and alternative ways of experiencing spirituality, such as virtual masses, many people do not feel the need for belonging or simply do not express their spiritual needs. (CC6)

I haven’t noticed that the virus has provoked a deeper search for God. Although it has unleashed existential fear, it hasn’t deepened spirituality. In fact, I think that many people feel comfortable with it [the remote pastoral care – JD] and will not come back to church. For some, it is easier to turn on the computer or watch the mass on TV. (EA)

However, some SCP believed that the pandemic has created new opportunities for pastoral and spiritual care. In particular, they emphasised that because COVID-19 has unleashed huge anxiety and fear, it has also increased the awareness of spiritual needs and a desire for faith, hope and trust in a better future that comes from religion. Moreover, they stressed that the pandemic has raised awareness of the role of SCP in the clinical setting.

[t]he pandemic has unleashed fear over oneself, one’s life and the health of one’s family. It has shown me that we have to develop a new model of spirituality that rests on faith, trust and hope in God protection, not on fear. Thus, it is faith and hope that should guide our actions not fear that paralyses us. (EA)

[h]ope that comes from the Bible (…) it is real source of relief. I think that it can be noticed especially during the pandemic, when the avalanche of bad news was even bigger. (JW1)

Finally, while describing themselves as “a God’s tool” (CC4), some hospital chaplains reflected how their presence has helped patients to acknowledge their spiritual needs or even regain their faith.

Especially during the first wave, I received many positive opinions that it has helped people get closer to God, they pray more often and have more time for themselves and their families. (CC12)

Even today, I had such a positive experience. I entered one room with four male patients and asked if they wanted to receive communion. They looked at me with disapproval, so I wanted to leave when one of them shyly requested the sacrament, and soon another also asked for it, and the third too. In the end, they all received it. (…) It was very positive … Such testimonies, when a person, especially male, admits to their faith and gives a public testimony, it helps me survive difficult moments. (CC1)

## Theme 7: The Role of Spiritual Care During the Outbreak

Even though the pandemic disrupted SCP’s usual practice and hindered their contacts with the patients and families, all participants emphasised the unique contribution of SCP to the healthcare system and strongly believed that their role was to provide a compassionate and supportive presence to all those who need it. They also declared that, especially at the time when no cure was available, the value of the holistic approach and spiritual care was essential.

Spiritual care is very needed and many people really desire it. (CC4).

Apart from the problems caused by the pandemic and the fact that it has influenced the way we provide care, it has increased the need for spiritual care. (CC7)

The pandemic made us feel even more important because it occurred so unexpectedly and we had to pay special attention to our fellow brothers. Thus, from the very beginning, we started caring about their mood, how they felt and what they needed; and it made us feel very important (…) The pandemic has encouraged us to be more vigilant (JW2).

While some Catholic priests emphasised the role of the sacraments, others suggested that it is the conversation about the final issues that matter the most. Yet others thought that spiritual care allows those who are isolated and overwhelmed by their sickness to gain a different perspective on the disease and suffering.

[f]or me the fundamental aspect of [spiritual – JD] care is providing the sick with the sacraments, whether penitence or the Eucharist. Frequently, when I enter the room and ask who wants to receive a sacrament it is everything to these people. They often do not need to talk. They have plenty of spare time to think and they have often managed to organise everything in their conscience. (CC1)

Spiritual service is very important. I am there with them. It gives them [patients – JD] courage. It allows them to have a good cry and to let it all out. It is all very calm; usually saying: ‘Father, it’s good you are here’. My presence helps them a lot. (CC6)

However, many others believed that spiritual care should not be focused on rituals or philosophical and theological discussions on the meaning of life and death. While stressing the therapeutic value of physical contact, face-to-face interaction and silent presence, they suggested that their role as SCP was simply to be with those who needed it.

Physical presence and personal contact are the most important aspects of spiritual care. It should not be all about rituals or sacraments. Of course, they are important, but it is our physical presence that is essential. It cannot be underestimated as simple being with a person, and the sick in particular, has a great therapeutic value. (EA)

I think that my main task as a chaplain is not to conduct philosophical discussions on the nature of life and death, the meaning of life or whether it is God who sends diseases; but to be next to the sick, so the patient could experience the closeness of God and understand that God is also suffering with him/her… the role of the priest is to be with people, assist them and give the testimony of the closeness of God, not to give simple answers but oneself. (CC12)

## Theme 8: The Healthcare Professionals’ Perceptions of SCP

Participants also reflected on how they were perceived by the healthcare professionals and what reactions they faced during their service. Most respondents believed that healthcare professionals are aware of the importance of spiritual care and perceive SCP as important members of healthcare teams. Additionally, they commented on how the clinicians helped them with the safety procedures or putting on the protective uniforms.

The personnel are very helpful. When I enter special wards where I have to wear not only a mask and protective helmet but also a safety uniform, someone always helps me with that. (CC1)

There were situations when some medics were surprised that I am in the hospital but basically they found it positive; they understand our presence on the wards. The personnel helps us a lot (…) there are many acts of kindness: I am being helped with putting on my protective uniform or being asked whether I knew all the procedures and if I needed any advice. (CC12)

Moreover, participants argued that it is often physicians or nurses who suggested that a chaplain should pay special attention to patients’ spiritual needs. Thus, although some SCP reflected on having problems with fitting in and finding oneself within the medical hierarchy, they stressed that neither the healthcare professionals nor the directors had ever treated them with any sense of superiority. On the contrary, while recognising their role as essential, the clinicians often acknowledged that spiritual care requires special knowledge, skills, charisma and internal motivation. Consequently, most SCP felt trusted, important and needed, and believed they were important members of the medical team.

I feel important. I’ve been treated by the staff as a full-fledged member of a therapeutic team and we simply try to help each other. (CC6)

I have never felt unnecessary or useless. On the contrary, each day, at every turn I see many positive attitudes from the medics. My role as a chaplain is not being unnoticed. Many health care professionals appreciate my presence and my work, and I often hear that I am very needed. I think that their studies have helped them to understand such needs. Every single nurse is sensitised, no matter whether she lives by the faith or not, they just know it and accept it. For us, the chaplains, this is of great help. They help us to recognise the situation. (CC11)

However, some participants complained that there were situations when either the directors or the personnel were reluctant bout their presence on the wards and did not understand the work they do for patients. While stressing how hard it is to implement spiritual care in the clinical setting, some SCP felt that it is two separate realities that do not understand each other: the one of pastoral/spiritual care and the world of medicine.

I feel like someone separate, I have my own field of activity but I do not feel as being a part of the healthcare team. (CC13)

Our work is often not recognised as important, and sometimes it is even neglected (…) healthcare professionals are focused on the physical dimension of disease and are often not interested in other dimensions of health. Thus, when a priest enters the ward he is a sign of a different reality. (…) I haven’t noticed that the pandemic has changed the attitude of the clinicians towards spiritual care. (CC17)

Although there is progress, still many health care professionals believe that spiritual care, if necessary at all, is something separate from medical care. Meanwhile, Christianity presents a holistic view on care that cannot be reduced to rituals or sacraments. It should be provided by trained professionals who operate spiritual language and are familiar with such concepts as the feeling of guilt, forgiveness, reconciliation with God, absolution or sin and who accompany a person in their searching for spirituality and spiritual problems. (EA)

## Theme 9: Barriers to the Provision of Spiritual Care During the Pandemic

Although the Polish Constitution warrants every citizen with the freedom of conscience and religion, some SCP suggested that the COVID-19 pandemic has resulted in the limitation of patients’ right to spiritual care. While some SCP were not allowed to enter the hospital at all, others were restricted to only visit some wards. Thus, while participants acknowledged that due to sanitary reasons personal contact with the patients may be limited, they stressed it cannot be prohibited, especially when it considers chaplains working as hospitals’ full-time employees. However, they argued that because healthcare professionals are focused on the disease itself and medical procedures, SCP are often restricted from visiting the wards. Moreover, some directors suggested that pastoral/spiritual care is not essential and should be limited or even cancelled during the pandemic as the patients’ spiritual needs may be fulfilled via television or radio.

I haven’t been to the hospital for a year. Although legally I have the right to visit patients in hospitals, there is a kind of informal regulation that ‘non-essential’ visitors, such as full-time chaplains, should not come. (EA)

There was even a time when we were not let into the chapel and were forbidden to celebrate the mass. It’s sad, but sometimes they make such difficulties. (CC3)

There was a danger that it all [spiritual care – JD] could be cancelled. They thought about terminating my service. The head physician recommended against or even prohibited me from visiting two wards. Still, I am not allowed to enter there. During a remote conference, the director suggested that I should minister all the sacraments ‘spiritually’ without my presence. It was a very difficult experience. I thought they will cancel entirely spiritual care. (CC4).

Other SCP complained that some healthcare practitioners were prejudiced and opposed their presence on the wards. Consequently, some participants felt marginalised and discriminated against and reflected that it was suggested to them that an SCP is little more than an ‘infection risk’ and poses a threat.

On one ward I felt like being subjected to mobbing as the staff was treating me like a ‘necessary evil’. I felt as if being disregarded or ignored. Sometimes they made difficulties with entering the ward, even though I had all permissions. (CC4)

At the beginning, some suggested that as a priest I shouldn’t enter the ward as I might infect someone. Yes, I was accused of spreading the plague. Such stigmatisation was very difficult. (CC6)

Finally, some chaplains complained that despite growing evidence for their positive impact, SCP were undervalued, misunderstood or even mocked. They felt sorry that while the healthcare professionals were applauded as heroes, SCP and hospital chaplains, in particular, were often neglected or perceived as useless. They also suggested that such negative attitudes reflected general prejudices towards religion and the church which escalated after the tightening of the abortion law.

Some clinicians, are very impolite and say that the priest is unnecessary or other offensive things. (…) Yeah, it happens, however, it is not surprising taking under consideration the current social protests related to abortion. (CC1)

Sometimes I feel sorry that while the media notice and inform the public about the heroic work done by the healthcare professionals, they avoid, like holy water, mentioning the priests. But we are also there, and we also do an important thing for patients. (CC14).

## Discussion and Conclusions

After a year of the COVID-19 pandemic, morbidity and mortality rates in Poland continue to mount resulting in an increased prevalence of stress, anxiety and depression in the general population (Bartoszek et al., 2020; Lahav, 2020; Malesza & Kaczmarek, 2021; Salari et al., 2020). At the same time, religion/spirituality has been associated with better mental health outcomes when dealing with stressful life events, including acute, infectious or chronic diseases (Hamilton et al., 2021; Pirutinsky et al., 2020; Shapiro et al., 2021; Sohail et al., 2020; Tarakeshwar et al., 2006; Thomas, & Barbato, 2020). However, due to various control measures imposed by the Polish government during the current health crisis, religious coping has been seriously limited.

Indeed, even though some faith-based communities resisted the efforts of the state agencies to limit religious gatherings (Singh, 2020; Williams et al., 2021), the lockdown following the first wave of the pandemic in Poland resulted in the temporal closing of all churches, banning of religious gatherings or limiting the number of people who could be physically present during services (Kowalczyk et al., 2020).

Thus, even though most congregations have developed alternative means of providing pastoral service, religious/spiritual life in its community-based dimension has been seriously restricted (Sułkowski & Ignatowski, 2020; O’Brien, 2020; Shapiro et al., 2021). Moreover, similar to other countries, during the second and the third waves of the pandemic to balance between safety and piety, some churches in Poland, i.e. Catholic and Orthodox, limited their religious life in their community-based dimension, while others, i.e. Protestant, have totally suspended it (Sułkowski & Ignatowski, 2020).

This is of key importance because religious/spiritual care is an integral part of the holistic approach and one of the domains of quality care. Thus, unsurprisingly, while adapting to the restrictions, religious congregations have tried to maintain contact with the believers, shifting towards online-based services, spiritual recollections and retreats, community prayers and sacraments (Sułkowski & Ignatowski, 2020; O’Brien, 2020; Shapiro et al., 2021). However, for many people, the sick in particular, religious/spiritual care is unavailable. Consequently, they are suffering and dying in isolation from their families, relatives and (religious) communities. While the transition from the physical to the virtual presence is highly needed, the coronavirus crisis has amplified the importance of spiritual care, especially for patients alone in hospitals (Dutra & Rocha, 2021; Harrison & Scarle, 2020; Krajnik et al., 2020; Papadopoulos et al., 2021; Peacock, 2020; Swift, 2020).

However, although the physical and mental health consequences of the SARS-CoV-2 are well described (Carfì et al., 2020; del Rio et al., 2020; Vindegaard & Benros, 2020), much less is known about spiritual needs and spiritual care during the COVID-19 pandemic. It is not surprising because while focussing on patients’ physical or mental health, healthcare professionals often neglect the spiritual dimension. Meanwhile, the importance of spiritual health and spiritual care in critical situations has long been recognised (Baldacchino, 2015; Carey & Mathisen, 2018; Fitch & Bartlett, 2019; Graves et al., 2002; Puchalski et al., 2014) and there is growing evidence for the positive role of SCP in the healthcare system during the current pandemic (Chirico & Nucera, 2020b; Koenig, 2020; Papadopoulos et al., 2021).

For example, Koenig (2020) emphasised the role of religious faith and practice in maintaining good health during the outbreak. Similarly, Cadell et al. (2021) showed that even in secular cultures, spirituality has played an active and positive role in the quality of life and health during the COVID-19 pandemic. Also, Ferrell et al. (2020) stressed that the COVID-19 has revealed an urgent need for spiritual care as an integral part of whole-person palliation. A similar observation has been made by Sarmiento (2020) who called for spiritual care for healthcare professionals during the COVID-19 pandemic. Additionally, Wierstra et al. (2020) demonstrated that the pandemic has increased the visibility of healthcare chaplains, strengthening their role in healthcare settings.

At the same time, Murphy (2020) showed that although the COVID-19 has brought forward many untimely and unexpected deaths, social isolation and distancing have negatively affected funeral rituals and bereaved relatives as many people were not able to properly grieve, it has also increased the chaplain’s role of supporting people through grief and loss. Peacock (2020) described how COVID-19 has negatively influenced the provision of spiritual care in the mental healthcare setting and stressed the need for new technologies in mental health chaplaincy. Swift (2020) demonstrated how the pandemic has changed the interactions of chaplains with the persons under their care from “being there” to “virtually being there” or even “being absent”.

Similarly, while Harrison and Scarle (2020) found that the nature of spiritual care had shifted towards telephone provision, Drummond and Carey (2020) showed that although the pandemic had negatively influenced Australian aged care, providing spiritual care from a distance can benefit both the elderly, their families, chaplains and healthcare organisations. Also, Busfield (2020) described how difficult and challenging it was for chaplains to maintain a calm presence during the first weeks of COVID-19. By contrast, Jones et al. (2020) showed that while chaplains played an important role for hospital patients, their families and healthcare staff, they also need to sustain their spiritual well-being.

Furthermore, this study confirms that while the COVID-19 crisis has seriously limited the possibilities of providing spiritual care and has transformed its nature, it has highlighted the unique contribution of SCP to the healthcare practice. During the current pandemic, many people were exposed to pain, fear and loneliness or experienced the feeling of near death, thus realising that pastoral/spiritual care in hospitals is essential and that people’s urgent spiritual needs should be fulfilled, especially in times of crisis (Chirico, & Nucera, 2020b; Dutra & Rocha, 2021; Hall, 2020; Heidari et al, 2020; Koenig, 2020; Krajnik et al., 2020; Papadopoulos et al., 2021; Pierce et al., 2020). It is of key importance because while healthcare professionals often stress the necessity of spiritual care, they do not possess the necessary skills to fulfil patients’ spiritual needs.

Thus, one must agree with Papadopoulos et al. (2021) that there is an urgent need to develop centralised strategies to prepare healthcare systems and professionals in relation to spiritual support provision, especially during health disasters and emergencies (Chirico & Nucera, 2020b). Similarly, Dutra and Rocha (2021) emphasise that while focussing on the sanitary measures that help to contain the spread of the virus, governments should not neglect promotion of mental and spiritual health that can minimise psychological damage in patients, their families and health professionals and should promote religious support programs in hospitals.

This is in accord with Weinberger-Litman et al. (2020) observation that by providing social support and promoting feelings of unity, religious institutions can play a vital role in supporting individuals’ psychological needs during a health crisis. Indeed, as the respondents interviewed emphasised, their role during the pandemic was not limited to addressing the pastoral, religious and spiritual needs of patients alone, but they were also present for their families and the staff. For that reason, despite the shortage of hospital chaplains and other SCP, there is a necessity for hospital authorities to create a supportive environment that could promote spiritual care in the clinical setting, providing a presence and space to meet the pastoral, religious and spiritual needs of all, especially in such desperate times (Chirico & Nucera, 2020b; Dutra & Rocha, 2021; Krajnik et al., 2020; Papadopoulos et al., 2021).

Moreover, there is also an urgent need to further incorporate SCP into healthcare teams, especially while society and the media do acknowledge the role of healthcare professionals in the fight against the pandemic, the role of SCP during the COVID-19 crisis is often an underrated aspect of caring. Indeed, apart from social distancing and other restrictions that disrupted the SCP’s usual practice, the most important barrier that hindered the delivery of spiritual care during the pandemic was the negative attitudes of the hospital authorities and healthcare professionals, who either did not understand what they did or did not believe that the spiritual care is an essential aspect of hospital care and opposed the presence of SCP on the wards.

Thus, there is also a need to increase the awareness of the importance of spiritual care among all healthcare professionals (Baldacchino, 2015; Graves et al., 2002; Krajnik et al., 2020; Puchalski et al., 2014; Tiew et al., 2013; Wu et al., 2016). Finally, this study confirms Snowden’s (2021) argument that while SCP understand their role during the pandemic, they are often confused about their position in the healthcare teams. For that reason, further research should focus on the collaboration of the SCP with healthcare professionals, their effective integration into the healthcare system as well as how such cooperation could benefit patients.

## Study Limitations

This study has some limitations. First, only twenty-four spiritual care practitioners were interviewed and the vast majority were Catholic priests. However, despite the small sample, it must be acknowledged that thematic saturation was achieved. Second, the study has a local dimension as only responses from SCP from one location were analysed. Consequently, it would be desirable to compare the findings from other locations in the country. Third, because six SCP refused to participate in the study, the results represent solely the opinions of those who agreed to participate in the study and cannot be generalised for the entire population of SCP either in Poznan or in Poland as a whole. Finally, as the entire analysis was performed by one author alone, there was a higher risk of subjectivity that might have influenced both the choice of the themes and the interpretation of the data.

However, despite these limitations, some advantages of this study should also be acknowledged. Most importantly, as there is a scarcity of previous work on the topic, this research fills the gap in the literature regarding the experiences of SCP facing the COVID-19 pandemic in Poland. Moreover, by providing new insights, it emphasises the role of spiritual care during the current health crisis. Finally, it enabled the SCP to share their experiences, which might have a therapeutic value.

## Appendix: Interview Questionnaire

Date:

Interviewer:

## Introduction

This is a questionnaire for the research on spiritual care practitioners’ experiences of the COVID-19 pandemic. I would like to elicit your view on the current healthcare crisis caused by the COVID-19 outbreak and the way it has affected the provision of spiritual care. At the same time, I wish to assure you that the results of these interviews will be collected anonymously and used solely for scientific purposes.

Gender……………………………………………………………………………………………

Age………………………………………………………………………………………………

Years of experience ……………………………………………………………………………..

Nationality……………………………………………………………………………………….

Location…………………………………………………………………………………………

Denomination……………………………………………………………………………………

Position ………………………………………………………………………………………….

Place of service ………………………………………………………………………………….

1. How long have you been working as a spiritual health practitioner/healthcare chaplain?
2. Do you serve in a hospital or a community setting?
3. How do you perceive your role as a spiritual care provider/healthcare chaplain?
4. What was your reaction when you heard about the COVID-19 pandemic and the governmental restrictions? How did you respond to this crisis?
5. What are your experiences from the pandemic? What do you find the most difficult? Do you see any positive aspects of the pandemic?
6. Has the pandemics affected the provision of spiritual care? What challenges do spiritual care providers/healthcare chaplains face during the COVID-19 pandemic?
7. What are patients’ spiritual needs during the COVID-19? Has the pandemic changed the character of those needs? What do you do for patients?
8. Do healthcare professionals ask for your spiritual guidance? What are their needs during this crisis?
9. Has the pandemic influenced your perception of life, dying and death?
10. How do you cope with the pandemic-related stress?
11. What reactions do you face while providing spiritual care during the coronavirus pandemic? How do the hospital authorities, healthcare professionals and patients react to your service?
12. Have you ever experienced any type of discrimination?
13. How does the pandemic affect the role of spiritual care and healthcare chaplaincy? Do you feel as if you are an integral part of the healthcare team? Do other healthcare professionals treat you as a member of the healthcare team?
